# The Relationship between Job Strain and Ischemic Heart Disease Mediated by Endothelial Dysfunction Markers and Imaging

**DOI:** 10.3390/medicina60071048

**Published:** 2024-06-26

**Authors:** Paloma Moisii, Irina Jari, Andra Mara Ursu, Alexandru Gratian Naum

**Affiliations:** 1 1st Medical Department, “Gr.T.Popa” University of Medicine and Pharmacy, 16 Universitatii Street, 700115 Iasi, Romania; 2 “Promedicanon” Cardiology Office, 15 Valea Prisacii, Valea Lupului, 707410 Iasi, Romania; 3 2nd Surgical Department, “Gr.T.Popa” University of Medicine and Pharmacy, 16 Universitatii Street, 700115 Iasi, Romania; 4 Radiology and Medical Imaging Clinique, “Sf.Spiridon” Emergency Hospital, 1st Independentei Avenue, 700111 Iasi, Romania; dr.andraursu@gmail.com; 5 2nd Morpho-Functional Department, Biophysics and Medical Physics, “Gr.T.Popa” University of Medicine and Pharmacy, 16 Universitatii Street, 700115 Iasi, Romania; alexandru.naum@gmail.com; 6 “Neolife” Medical Center, 52 Carol I Avenue, 700503 Iasi, Romania

**Keywords:** coronary heart disease, cardiovascular risk, job strain, high-sensitivity C-reactive protein, albumin urinary excretion rate

## Abstract

*Background and Objectives*: Job strain is a psychological, physical, and behavioral stress that occurs at the workplace. Job strain is associated with more than double the normal risk of coronary artery disease (CAD). The main aim of this study was to determine the association between job strain and the following parameters: high-sensitivity C-reactive protein (hs-CRP), the albumin urine excretion rate (AUER), and secondary-level testing. *Materials and Methods*: This study was a descriptive cross-sectional study conducted on patients who underwent cardiological assessment between October 2023 and February 2024 at the Promedicanon Cardiology Center. This study comprised 210 participants, with two groups: 105 chronic coronary syndromes (CCS) patients and 105 no-CCS patients. The baseline characteristics collected were age, gender, education, rural/urban environment, traditional CAD risk factors, hs-CRP, and AUER. The secondary-level testing included an electrocardiogram (ECG), echocardiography, and enhanced contrast computed tomography (ECCT). Psychological questionnaires comprised the tertiary-level testing, including the PHQ-9 depression questionnaire, and the satisfaction with work scale (SWWS) for job strain (Likert score). *Results*: The baseline characteristics were all significantly different between the groups (*p* < 0.05) except for total cholesterol. The hs-CRP level had a mean value of 0.4837 ± 0.19082 in the CCS group; for the no-CCS group, the hs-CRP mean value was 0.2289 ± 0.11009; *p*-value < 0.001. The AUER had a mean value of 42.770 ± 12.8658 for the CCS group and 26.432 ± 9.7338 for the no-CCS group; *p*-value < 0.001. For the associations between secondary-level testing and job strain: *p* < 0.001 for ST depression, negative T-waves, and q-waves; *p* = 0.415 for atrial fibrillation (AF); *p* = 0.018 for wall motion studies; *p* = 0.005 for ECCT. The association between job strain and AF had no statistical significance. The contractility of left ventricle walls and coronary calcification score were associated with job strain, with statistical significance. The *p*-value was 0.013 for the relationship between depression and the ECCT; for the association between depression and CCS status, the *p*-value was 0.021. Depression is usually diagnosed in job strain. The association between depression, and coronary calcification, as well as depression and CCS status had statistical significance. *Conclusions:* Job strain increased the hs-CRP level and AUER in both the CCS and no-CCS patients. The primary and secondary prevention of CHD could also include interventions to reduce job strain.

## 1. Introduction

Chronic coronary syndrome (CCS) and acute coronary syndromes cause 29% of deaths (World Health Organization 2023). For a century, we considered CAD to be provoked by an obstructive atheromatous plaque inside a large coronary artery. Then, a new concept was defined: nonobstructive coronary CAD or open artery ischemia (OAI). Patients with this condition form the majority of CAD patients. The mechanisms of OAI are a hypercoagulable status, non-obstructive atheromatous plaque, and coronary microvascular dysfunction. New clinical concepts were also defined: angina with no obstructive CAD (ANOCA), ischemia with no obstructive CAD (INOCA), and myocardial infarction with no obstructive coronary arteries (MINOCA). OAI is more frequently diagnosed in women in particular [1]. 

Job strain is an imbalance between heavy efforts (physical and/or psychological) and rewards; it frequently occurs if this imbalance is associated with short time, for high demands and/or with short time for relaxation, during the day or the year. Job strain promotes and worsens CAD and alters the quality of life among patients with acute coronary syndrome [2] and chronic CAD [3]. During this type of stress, hs-CRP and inflammatory cytokines are released in the bloodstream. In young and healthy subjects with sustained job strain, an elevated hs-CRP can lead to CAD [4]. Lower levels of leisure time and physical activity are correlated with higher levels of hs-CRP, especially in people with job strain. Worldwide, sedentariness is also a problem related to CAD. Physical activity can decrease hs-CRP levels and CAD risk [5]. The atherosclerotic process is connected with hs-CRP, as this biomarker stimulates the uptake of LDL cholesterol into macrophages [6]. Hs-CRP is also implicated in cardiovascular outcomes [7,8].

The AUER is a marker of endothelial dysfunction, which may lead to increased atherogenic status [9]. An elevated AUER is associated with subclinical atherosclerosis [10]. Subjects employed in shift work have increased AUER values [11]. In addition, subjects with job strain present elevated AUER values, compared with subjects without job strain, independent of CAD risk factors [12].

HsCRP, AUER, and traditional cardiovascular risk factors, like lipid profile, glycemia, are the baseline testing in CAD. Second-level testing in CAD includes the following: electrocardiogram (at rest or exercise test), transthoracic echocardiogram, enhanced contrast computed tomography, computed tomography coronary angiogram, and standard coronary angiogram.

An unhealthy lifestyle may substantially increase the CAD risk among people with high job strain [13,14]. Imbalanced effort–reward and sustained stressful situations in the workplace have implications for CAD outcomes [3]. Psychosocial stressors at work also increase CAD risk [2,15].

Our research had the following objectives, illustrated in Table 1.

## 2. Materials and Methods

### 2.1. Study Design

This work was a descriptive cross-sectional study, conducted between October 2023 and February 2024. The study involved 210 participants recruited through the Promedicanon Cardiology Center. The participants were living and working in 7 counties of Romania: Iasi, Neamt, Botosani, Suceava, Bacau, Galati, and Vrancea.

### 2.2. Research Samples

The participants formed 2 distinct samples as follows: one experimental group with CCS, including angina pectoris (AP), silent myocardial ischemia (SMI), and chronic myocardial infarction (CMI), had 105 participants; and the second group (control group), without CCS, had 105 participants, with traditional cardiovascular risk factors (smoking/obesity/hypercholesterolemia/arterial hypertension/diabetes mellitus). The subjects were referred to a cardiologist by a general practitioner (GP) for cardiovascular risk assessment.

Any participant could discontinue participation at any point during the research. The data collection ensured patients’ anonymity. The demographic characteristics of the patients were collected, including age, gender, and rural/urban environment; in addition, we specified the education level: elementary school, secondary (high) school, or higher education. The inclusion criteria for both samples are summarized in Table 2.

The exclusion criteria are illustrated in Table 3.

### 2.3. Methods

We utilized the following methods for all of the patients:

1. A complete clinical examination;
2. An ECG, conducted using Heart Screen 80 G (Innomed Medical, Budapest, Hungary);
3. A transthoracic echocardiogram, conducted using a Fukuda Denshi 850 XTD (Fukuda Denshi in Tokyo, Japan);
4. An ECCT, only for CCS patients. The Agatston ECCT score describes the severity of coronary artery calcification, as detailed in Table 4;

5. Lipid profile, glycemia, hs-CRP, and AUER, determined by chemistry laboratories;
6. PHQ-9 questionnaire [17,18]. The PHQ-9 is a psychological tool for depression assessment. The test has nine specific questions. The level of depression is considered mild if the PHQ-9 score is 5–9. These patients should repeat the test after 1 month. The same PHQ-9 score, after 6 months, requires counseling. Moderate (PHQ-9 score = 10–14) and moderately severe (PHQ-9 score = 15–19) depression require counseling and medication. Severe depression (PHQ-9 score) requires immediate and active psychiatric intervention;
7. SWWS questionnaire; see Blais et al. [19]. The SWWS is a psychological questionnaire with 5 items as affirmations. These affirmations are illustrated in Table 5:

All 5 items of the SWWS (a, b, c, d, e) express satisfaction with the workplace. Every participant received this questionnaire during the medical conversation. The subject had to choose an answer on a five-point scale from ‘’totally disagree” to “totally agree” for each affirmation. The five-point Likert score for each answer was recorded by the clinician, as shown in Table 6. The worst Likert score was 1, which corresponded to “totally disagree”. These subjects had severe dissatisfaction with their workplace. The best Likert score was 5, which corresponded to “totally agree”. These subjects had high satisfaction with their workplace. Likert scores from 2 to 3 to 4 showed a growing level of satisfaction with the workplace.

This study was approved by the Ethical Committee for Research, 351/9 October 2023, from “Gr.T.Popa” University of Medicine and Pharmacy, Iasi, Romania, www.umfiasi.ro.

### 2.4. Statistical Analysis

The Pearson Chi-square test was used to compare the baseline characteristics of the CCS and no-CCS groups. We also utilized the Chi-square test for the following associations: Likert score and CCS status, Likert score and EKG/wall motion studies on echocardiography/Agatston ECCT score, depression severity and ECCT score, depression severity and CCS status. The ANOVA test was utilized for the following associations: Likert score and hs-CRP and AUER. After stratifying the population into the CCS and no-CCS groups, we verified through ANOVA whether job strain had a link with hs-CRP and AUER in one or both groups.

The statistical analysis software used was SPSS 29. A *p*-value of <0.05 was considered statistically significant.

## 3. Results

### 3.1. Baseline Characteristics of the Groups (CCS and No-CCS)

The median age of the CCS patients was 69 years. The no-CCS patients were younger than the CCS patients, with a median age of 52 years. In addition, female patients were predominant in the no-CCS group. A high school education was predominant in both groups. Among the traditional cardiovascular risk factors, obesity and arterial hypertension had the highest prevalence in both groups. The *p*-value showed statistical significance for each of these baseline characteristics. Only the total cholesterol was not statistically significant. Table 7 illustrates these results.

The hs-CRP level and AUER were the baseline markers for endothelial dysfunction. We tested the association between the Likert score and hs-CRP/AUER. We noticed a highly statistically significant association between the Likert score and these two variables of interest for the global sample. Table 8 presents these results.

The same highly statistically significant association between hs-CRP/AUER and job strain severity was also identified separately in the CCS and no-CCS groups. Table 9 and Table 10 summarize these results.

The hs-CRP level and AUER showed elevated mean values for the global sample because they are endothelial dysfunction markers. The mean values were higher in the CCS group than in the no-CCS group. These results suggest the specific impact of the hs-CRP and AUER in cardiovascular risk stratification.

### 3.2. Secondary-Level Testing Results: EKG, Echocardiography, and ECCT

ST depression and negative T-waves, as well as q-waves, were correlated with job strain severity, with high statistical significance. However, the association between AF and the Likert score had no statistical significance. Table 11 illustrates these results.

The association between the wall motion studies and job strain was statistically significant. These results are shown in Table 12.

The association between the ECCT score and job strain was highly statistically significant, as illustrated in Table 13.

The Agatston score was stratified by depression status, with statistical significance. These results are shown in Table 14.

For secondary-level testing, the relationship between ST depression, negative T-waves, q-waves on the EKG, and job strain was highly statistically significant, as was the association between the ECCT score and job strain. AF had no statistically significant association with job strain. The association between the wall motion studies on the echocardiography and job strain was statistically significant. All of these results are summarized in Table 15.

### 3.3. Questionnaire Results

Depression was associated with CCS status, with statistical significance. Table 16 illustrates this association.

The association between the Likert score and CCS status had no statistical significance, which is explained by the fact that the no-CCS patients are not healthy people, as they have traditional cardiovascular risk factors. They are patients with obesity, arterial hypertension, diabetes melitus, and hypercholesterolemia. Table 17 illustrates these results.

## 4. Discussion

In our study, we had a predominance of elderly patients in the CCS group (mean age = 69 years). Age is the strongest risk factor for CAD [20]. The U.S. aged population is predicted to grow in future decades [21,22,23]; the same is true in Romania. The no-CCS patients were younger than the CCS patients (mean age = 52 years). They were diagnosed early as having CAD risk factors by a GP. A GP usually examines all of the relatives (young, middle-aged, and older) of a patient with CAD, and refers the patients with CAD risk factors to a cardiologist.

Female patients comprised the majority in both groups, CCS and no-CCS. Increased job and social strain among women leads to a higher prevalence of arterial hypertension, diabetes melitus, and CAD. This was also observed by authors such as Wang, Vogel, and Hossieni [24,25,26]. Premature CAD mortality was also reported in women by other authors [27,28].

The education level was high school for more than half of our patients. These patients, with a middle level of education, understood the utility of lifestyle changes, sustained treatment, and regular follow-up. In a large study focused on Eastern Europe, the authors noticed a remarkably high burden of coronary artery disease (CAD) in the northern parts of Eastern Europe. Romania is located in Central–Eastern Europe. According to the previously mentioned study, Romania had intermediate levels of CAD incidence and mortality rates. Poland (located in Central–Eastern Europe) had the lowest rates, and Ukraine (North–Eastern Europe) had the highest rates. The authors explained these disparities as being due to the different income and education levels and prevalences of cardiovascular risk factors in different Eastern European countries [29].

Medical education for patients at risk of CAD is another aspect. It is usually provided by doctors. In the future, nurses, clinical pharmacists, and nutritionists could also offer medical education on how to control cardiovascular risk factors, as suggested by Brown et al. [30].

LDL cholesterol was statistically significantly different between the two groups in our study. Testing for LDL cholesterol enables treatment to be modulated after a cardiovascular event [31,32]. Patients in primary prevention (like the no-CCS sample in our study) require personalized strategies to diminish their cardiovascular risk [33].

Severe dissatisfaction with the workplace was correlated with elevated levels of hs-CRP in our study. This observation was identified for the global sample and for each group separately. These results suggest that the hs-CRP level can be utilized in job strain assessment, for subjects with myocardial ischemia, and subjects with CAD risk factors.

The hs-CRP level is a reflection of the inflammatory status and, potentially, atherosclerosis [34]. Job strain provokes the release of inflammatory cytokines, followed by elevated hs-CRP values [35,36]. Atherosclerotic plaque progression is strongly correlated with the hs-CRP level [37].

In our study, the AUER showed elevated values in association with high job strain. Like for hs-CRP, this observation was identified for the global sample and for each group separately. These results suggest that the AUER can be utilized in job strain assessment for the primary or secondary prevention of CAD. The AUER is a marker of extensive endothelial dysfunction and is an atherosclerotic risk factor, especially in diabetes mellitus and arterial hypertension [38]. However, the AUER is an underappreciated risk factor for CAD and other vascular diseases [39], and it is an important parameter for cardiovascular risk prediction and the prevention of cardiovascular events [40].

The CCS group also included patients without obstructive coronary artery stenosis (OAI: ANOCA/INOCA/MINOCA). OAI is predominant in women. Depression and distress have an impact on OAI. In our study, the EKG, wall motion studies, and CT scores were correlated with job strain. These non-invasive tests were useful in our study, as women were the majority. In addition, we noticed that depression severity was correlated with the CCS status and Agatston ECCT score.

Many studies have detailed the connection between depression and atheromatous plaque. Somatic symptoms in depression predict cardiovascular events, and all-cause mortality. Cognitive symptoms in depression are not associated with this prediction [41]. In our study, we noticed that extensive calcification of coronary arteries was frequently noticed in severe depression. And, Agastson score is a reliable indicator of CAD severity [42]. Dysfunction of the autonomic nervous system leads to chronic inflammation, and chronic inflammation promotes atherosclerosis. Depression provokes low-grade inflammation, with negative impact towards the endothelium [43]. In our study, low-grade inflammation was quantified by hsCRP values. The patients with severe dissatisfaction with their workplace registered severe depression, and high levels of hs CRP. Negative emotions, such as anger, affect endothelium-dependent vasodilation. Reactive hyperemia and specific microparticles which are produced by the endothelium can be measured. Anger is associated with impaired vasodilation and high levels of these microparticles. And anger is frequently noticed in depression [44]. In our study, we noticed that severe depression repressed anger and social inhibition (difficult expression of personal thinking). These behaviors are specific for type D (“distressed”) personality and are associated with severe ischemic heart disease [45].

Our study results have some clinical implications. Baseline measurements of the hs-CRP level and AUER, for CCS and no-CCS patients, can be used to stratify the cardiovascular risk. The SWWS questionnaire can be used to assess the job strain level. The PHQ-9 questionnaire can determine the severity of depression. Interventions can be medical, interventional, and/or surgical. Psychotherapy and/or psychiatric medication can be applied as personalized strategies. After 3 months of treatment, a laboratory follow-up on the hs-CRP level and AUER can be used to evaluate the treatment’s efficacy (with a decrease in the hs-CRP level and AUER indicating a lower cardiovascular risk than the baseline levels).

One limitation of our study is the lack of job strain-related long-term effects towards physical and emotional health in CCS and no-CCS patients. Another limitation is the lack of comparison between different categories of vascular patients with job strain, i.e., peripheral artery disease, transient ischemic attack, ischemic/hemorrhagic stroke, ischemic heart disease.

Future directions for our research will assess the association between couples’ satisfaction, and CCS.

## 5. Conclusions

Dissatisfaction with the workplace induces elevated levels of the endothelial dysfunction markers hs-CRP and AUER. Secondary-level testing (ECG, echocardiography, and ECCT) can confirm the CCS status, while questionnaires can be used to elucidate a patient’s job strain and depression levels. Specific interventions in CCS can thus combine cardiovascular and psychiatric tools. For CAD prevention (no-CCS patients, with arterial hypertension/diabetes melitus/hypercholesterolemia), the hs-CRP level and AUER can also help determine which interventions are appropriate.

## Figures and Tables

**Table 1:** The study’s objectives.

Objective	Description
1	Establish the prevalence of job distress in CCS patients compared with no-CCS patients.
2	Discover the role of hs-CRP and AUER as possible mediators in this relationship.
3	Determine the association between secondary-level testing and job strain.

**Table 2:** Inclusion criteria.

Inclusion Criteria	
1	Age ≥ 18 years
2	Informed consent and consent for publication
3	CCS diagnosis/no-CCS diagnosis with cardiovascular risk factors: smoking/obesity/hypercholesterolemia/arterial hypertension/diabetes mellitus.

**Table 3:** Exclusion criteria.

Exclusion Criteria	
1	Progressive cancer
2	Autoimmune disorder
3	Pregnancy
4	Difficult transportation to cardiology center
5	Acute myocardial infarction
6	Unstable angina pectoris (de novo/worsened)

**Table 4:** The severity of coronary artery calcification [16].

Scoring	Interpretation
0	No measurable calcified plaque
1–10	Minimal
11–100	Mild
100–400	Moderate
>400	Extensive

**Table 5:** Satisfaction with work scale (SWWS): affirmations [19].

Affirmation
a. “Generally speaking, my work corresponds with what I want in my life”
b. “My working conditions are excellent”
c. “I am satisfied with my work”
d. “I have achieved things important to me at work, until now”
e. “If I could change something at my workplace, I wouldn’t change anything”

**Table 6:** Likert scores for the SWWS questionnaire [19].

Likert Score	Patient’s Answer
1	“totally disagree”
2	“partially disagree”
3	“almost agree”
4	“agree”
5	“totally agree”

**Table 7:** Baseline characteristics of the samples.

Characteristics	Total (210)	CCS (105)	No-CCS (105)	*p* Value
Age, years, median (IQR)	60 (22)	69 (16)	52 (18)	0.01
Sex, female, n (%)	130 (61.9)	58 (55.2)	74 (70.5)	0.03
Education n (%)	1. Elementary school 42 (20) 2. High school 128 (61) 3. Higher education 40 (19)	1. Elementary school 28 (26.6) 2. High school 59 (56.2) 3. Higher education 18 (17.2)	1. Elementary school 14 (13.3) 2. High school 69 (65.7) 2. Higher education 22 (21)	0.04
Total cholesterol (mg/dL) median (IQR)	248 (55)	260 (59)	246 (52)	0.16
LDL cholesterol (mg/dL) median (IQR)	182 (61)	186 (52)	177.5 (59.75)	0.03
Smoking n (%)	50 (23.8)	21 (20)	29 (27.6)	0.032
Obesity n (%)	124 (59)	66 (62.8)	58 (55.2)	0.04
Arterial hypertension n (%)	119 (56.6)	67 (63.8)	52 (49.5)	0.03
Diabetes mellitus n (%)	77 (36.6)	43 (41)	34 (32.4)	0.02

**Table 8:** Job strain, AUER, and hs-CRP for the global sample.

		AUER/24 h		hs-CRP	
Likert Score	n	Mean ± SD	ANOVA Test	Mean ± SD	ANOVA Test
1	35	50.943 ± 11.0302	*p* < 0.001 **	0.6260 ± 0.20291	*p* < 0.001 **
2	52	42.917 ± 10.6151		0.4283 ± 0.16049	
3	46	31.546 ± 8.4571		0.3135 ± 0.10382	
4	50	25.014 ± 7.6477		0.2270 ± 0.08981	
5	27	20.363 ± 8.0228		0.1804 ± 0.10607	
Total	210	34.601 ± 14.0203		0.3563 ± 0.20116	

n—number of patients; SD = standard deviation; ** = high statistical significance.

**Table 9:** Job strain and hs-CRP in the CCS and no-CCS groups.

Likert Score	n	hs-CRP				
		CCS			no CCS	
		Mean ± SD	ANOVA Test	n	Mean ± SD	ANOVA Test
1	22	0.7477 ± 0.14105	*p* < 0.001 **	13	0.4200 ± 0.09327	*p* < 0.001 **
2	28	0.5489 ± 0.11561		24	0.2875 ± 0.05495	
3	21	0.4105 ± 0.05714		25	0.2320 ± 0.04839	
4	20	0.3245 ± 0.04936		30	0.1620 ± 0.03326	
5	14	0.2757 ± 0.03797		13	0.0777 ± 0.02803	
Total	105	0.4837 ± 0.19082		105	0.2289 ± 0.11009	

n—number of patients; SD = standard deviation; ** = high statistical significance.

**Table 10:** Job strain and AUER in the CCS and no-CCS groups.

Likert Score	n	AUER/24 h			
		CCS		no CCS	
		Mean ± SD	ANOVA Test	Mean ± SD	ANOVA Test
1	35	57.200 ± 7.8104	*p* < 0.001 **	40.354 ± 6.6866	*p* < 0.001 **
2	52	50.104 ± 8.6739		34.533 ± 5.0315	
3	46	38.719 ± 6.7456		25.520 ± 3.6522	
4	50	31.915 ± 5.1729		20.413 ± 5.1528	
5	27	27.014 ± 4.1203		13.200 ± 3.7240	
Total	210	42.770 ± 12.8658		26.432 ± 9.7338	

n—number of patients; SD = standard deviation; ** = high statistical significance.

**Table 11:** EKG and job strain.

Likert Score	ST Depression and Negative T-Waves				Total		Pearson Chi-Square Test
	Absent		Present				
	n	%	n	%	n	%	
1	16	30.8%	6	11.3%	22	21.0%	Chi^2^ = 19.928
2	19	36.5%	9	17.0%	28	26.7%	*p* < 0.001 **
3	10	19.2%	11	20.8%	21	20.0%	
4	4	7.7%	16	30.2%	20	19.0%	
5	3	5.8%	11	20.8%	14	13.3%	
Total	52	100.0%	53	100.0%	105	100.0%	
Likert score	q-waves				Total		Pearson Chi-Square Test
	Absent		Present				
	n	%	n	%	n	%	
1	6	11.3%	16	30.8%	22	21.0%	Chi^2^ = 19.928
2	9	17.0%	19	36.5%	28	26.7%	*p* < 0.001 **
3	11	20.8%	10	19.2%	21	20.0%	
4	16	30.2%	4	7.7%	20	19.0%	
5	11	20.8%	3	5.8%	14	13.3%	
Total	53	100.0%	52	100.0%	105	100.0%	
Likert score	AF				Total		Pearson Chi-Square Test
	Absent		Present				
	n	%	n	%	n	%	
1	17	18.3%	5	41.7%	22	21.0%	Chi^2^ = 3.935
2	25	26.9%	3	25.0%	28	26.7%	*p* = 0.415
3	19	20.4%	2	16.7%	21	20.0%	
4	19	20.4%	1	8.3%	20	19.0%	
5	13	14.0%	1	8.3%	14	13.3%	
Total	93	100.0%	12	100.0%	105	100.0%	

n = number of patients; ** = high statistical significance.

**Table 12:** Job strain and ventricular wall motion findings.

Likert Score									Total		Pearson Chi-Square Test
	Normokinesia		Hypokinesia		Akinesia		Dyskinesia				
	n	%	n	%	n	%	n	%	n	%	
1	1	6.3%	5	12.5%	6	30.0%	10	34.5%	22	21.0%	Chi^2^ = 24.391
2	3	18.8%	7	17.5%	8	40.0%	10	34.5%	28	26.7%	*p* = 0.018 *
3	4	25.0%	8	20.0%	3	15.0%	6	20.7%	21	20.0%	
4	6	37.5%	10	25.0%	2	10.0%	2	6.9%	20	19.0%	
5	2	12.5%	10	25.0%	1	5.0%	1	3.4%	14	13.3%	
Total	16	100.0%	40	100.0%	20	100.0%	29	100.0%	105	100.0%	

n = number of patients; * = statistical significance.

**Table 13:** Agatston ECCT score and job strain.

Likert Score									Total		Pearson Chi-Square Test
	Extensive Calcification		Moderate Calcification		Mild Calcification		Minimal Calcification				
	n	%	n	%	n	%	n	%	n	%	
1	14	29.8%	5	25.0%	2	8.7%	1	6.7%	22	21.0%	Chi^2^ = 28.050
2	18	38.3%	6	30.0%	2	8.7%	2	13.3%	28	26.7%	*p* = 0.005 **
3	9	19.1%	4	20.0%	5	21.7%	3	20.0%	21	20.0%	
4	5	10.6%	3	15.0%	7	30.4%	5	33.3%	20	19.0%	
5	1	2.1%	2	10.0%	7	30.4%	4	26.7%	14	13.3%	
Total	47	100.0%	20	100.0%	23	100.0%	15	100.0%	105	100.0%	

n—number of patients; ** = high statistical significance.

**Table 14:** ECCT score and depression status.

Depression Severity									Total		Pearson Chi-Square Test
	Extensive Calcification		Moderate Calcification		Mild Calcification		Minimal Calcification				
	n	%	n	%	n	%	n	%	n	%	
Mild	3	6.4%	3	15.0%	5	21.7%	6	40%	17	16.2%	Chi^2^ = 20.917
Moderate	9	19.1%	2	10.0%	5	21.7%	6	40%	22	20.9%	*p* = 0.013 *
Moderate–severe	14	29.8%	7	35.0%	6	26.1%	2	13.3%	29	27.6%	
Severe	21	44.7%	8	40.0%	7	30.4%	1	6.7%	37	35.3%	
Total	47	100.0%	20	100.0%	23	100.0%	15	100.0%	105	100.0%	

n = number of patients; * = statistical significance.

**Table 15:** Secondary-level testing and job strain.

Statistical Significance of the Association					
ST depression	negative T-waves	q-waves	AF	Echo	ECCT
Job strain					
(Ls)					
**	**	**	no	*	**

Ls = Likert score; Echo = wall motion studies on echocardiography; * = statistical significance; ** = high statistical significance.

**Table 16:** Depression and CCS status.

		Sample				Total		Pearson Chi-Square Test
		CCS		no-CCS				
	DS	N	%	n	%	n	%	
	Mild	17	16.2%	39	37.1%	56	26.7%	Chi^2^ = 26.482
	Moderate	22	20.9%	37	35.2%	59	28%	*p* = 0.021 *
	MS	29	27.6%	17	16.2%	46	21.9%	
	Severe	37	35.3%	12	11.5%	49	23.3%	
Total		105	100.0%	105	100%	210	100%	

DS = depression severity; n = number of patients; * = statistical significance; MS = Moderate-Severe.

**Table 17:** Job strain and CCS status.

		Sample				Total		Pearson Chi-Square Test
		CCS		no-CCS				
	Likert score	N	%	n	%	N	%	
	1	22	21.0%	13	12.4%	35	16.7%	Chi^2^ = 5.007
	2	28	26.7%	24	22.9%	52	24.8%	*p* = 0.287
	3	21	20.0%	25	23.8%	46	21.9%	
	4	20	19.0%	30	28.6%	50	23.8%	
	5	14	13.3%	13	12.4%	27	12.9%	
Total		105	100.0%	105	100.0%	210	100.0%	

n = number of patients.

## Data Availability

The original contributions presented in the study are included in the article, further inquiries can be directed to the corresponding authors.
